# Design and implementation of a decision aid for juvenile idiopathic arthritis medication choices

**DOI:** 10.1186/s12969-017-0177-x

**Published:** 2017-06-05

**Authors:** William B. Brinkman, Ellen A. Lipstein, Janalee Taylor, Pamela J. Schoettker, Katherine Naylor, Karla Jones, Sheetal S. Vora, Catherine C. Mims, Elizabeth Roth-Wojcicki, Beth Gottlieb, Nancy Griffin, Carole Lannon, Esi Morgan

**Affiliations:** 1 Department of Pediatrics, Cincinnati Children’s Hospital Medical Center, 3333 Burnet Avenue, Cincinnati, OH 45229 USA; 2 University of Cincinnati College of Design, Architecture, Art and Planning, 5470 Aronoff, Cincinnati, OH 45221 USA; 3 Nationwide Children’s Hospital, 700 Children’s Drive, Columbus, OH 43205 USA; 4 Levine Children’s Hospital, 1000 Blythe Blvd, Charlotte, NC 28203 USA; 5 Medical University of South Carolina, 171 Ashley Ave, Charleston, SC 29425 USA; 6 Medical College of Wisconsin, 8701 W Watertown Plank Rd, Milwaukee, WI 53226 USA; 7 Cohen Children’s Medical Center of New York, 269-1 76th Ave, Queens, NY 11040 USA

**Keywords:** Juvenile idiopathic arthritis, Patient education, Shared decision-making, Decision aids

## Abstract

**Background:**

Randomized trials have demonstrated the efficacy of patient decision aids to facilitate shared decision making in clinical situations with multiple medically reasonable options for treatment. However, little is known about how best to implement these tools into routine clinical practice. In addition, reliable implementation of decision aids has been elusive and spread within pediatrics has been slow. We sought to develop and reliably implement a decision aid for treatment of children with juvenile idiopathic arthritis.

**Methods:**

To design our decision aid, we partnered with patient, parent, and clinician stakeholders from the Pediatric Rheumatology Care and Outcomes Improvement Network. Six sites volunteered to use quality improvement methods to implement the decision aid. Four of these sites collected parent surveys following visits to assess outcomes. Parents reported on clinician use of the decision aid and the amount of shared decision making and uncertainty they experienced. We used chi-square tests to compare eligible visits with and without use of the decision aid on the experience of shared decision making and uncertainty.

**Results:**

After 18 rounds of testing and revision, stakeholders approved the decision aid design for regular use. Qualitative feedback from end-users was positive. During the implementation project, the decision aid was used in 35% of visits where starting or switching medication was discussed. Clinicians used the decision aid as intended in 68% of these visits. The vast majority of parents reported high levels of shared decision making following visits with (64/76 = 84%) and without (80/95 = 84%) use of the decision aid (*p* = 1). Similarly, the vast majority of parents reported no uncertainty following visits with (74/76 = 97%) and without (91/95 = 96%) use of the decision aid (*p* = 0.58).

**Conclusions:**

Although user acceptability of the decision aid was high, reliable implementation in routine clinical care proved challenging. Our parsimonious approach to outcome assessment failed to detect a difference between visits with and without use of our aid. Innovative approaches are needed to facilitate use of decision aids and the assessment of outcomes.

## Background

Juvenile idiopathic arthritis (JIA) is the most common form of arthritis in children and adolescents, affecting approximately 1 in every 1000 young people. This chronic inflammatory disease is characterized by persistent joint inflammation that results in joint pain and swelling and restricted joint range of motion [1]. Several new, very effective drugs for JIA have been developed in recent years [2]. Because these medications differ in a variety of important attributes, such as mechanism of action, dosing interval, mode of administration, safety profile, and cost, some patients and families struggle to make treatment decisions and seek wide-ranging information from multiple sources [3]. The challenging process of obtaining enough information to make decisions can leave parents with long-lasting questions and concerns [4]. There is a need for improved communication with rheumatology clinicians about treatment decisions.

Decisions with multiple reasonable options that differ in ways that matter to families are conducive to shared decision making (SDM). SDM is a best practice in patient-centered care [5] and a recognized method to translate comparative effectiveness data into practice [6] and decrease unwarranted variation in healthcare [7]. In the SDM process, clinicians share information about treatment options and patients/parents share information about their goals and preferences. Working together, a treatment plan is developed that is the best fit for the individual patient and their family.

Decision aids, such as issue cards [8], are one way to implement SDM in practice by making decisions explicit and providing information about treatment options and their associated outcomes. Issue cards are designed to enable clinicians and patients/parents to efficiently discuss medications when there are multiple reasonable options that differ on a variety of attributes in a given clinical context [8]. Pioneered at the Mayo clinic [8], the issue card format has consistently led to patients/parents that are better informed and more involved in decision making in a variety of clinical contexts, including adults with rheumatoid arthritis [9–13].

However, while randomized trials have demonstrated the efficacy of patient decision aids [9, 14, 15], little is known about how best to implement these tools into routine clinical practice [16]. In addition, reliable implementation of SDM with decision aids has been elusive [17] and spread within pediatrics has been slow [18]. Commonly cited barriers to use of decision aids include time constraints, the absence of a reliable way to identify patients before decisions are made, the perception of too many educational materials, and lack of applicability due to patient characteristics and clinical situations [13, 19, 20].

Learning networks use the creative energies of patients, families, clinicians, and researchers to accelerate innovation, discovery and the application of new knowledge [21]. These learning health systems [22, 23] use continuing education and adult learning principles and an adaptation of the Breakthrough Series model [24] to guide improvement. The Breakthrough Series model promotes collaborative learning methods to understand and evaluate the issues, and begin testing changes that can help an organization make breakthrough improvements in the quality and value of health care [25]. The robust format of learning networks provides the infrastructure needed to understand and address the challenges of implementing SDM.

The Pediatric Rheumatology Care and Outcomes Improvement Network (PR-COIN) is a learning network launched in 2011 to improve outcomes of JIA care using quality improvement approaches [26, 27]. We designed a two-stage quality improvement project within PR-COIN to develop and reliably implement a decision aid to facilitate SDM between clinicians, patients with JIA, and their families around medication choices for treatment of inflammatory arthritis.

## Methods

### Setting

The project was conducted within PR-COIN during 2012–2014. At that time, the new and growing network consisted of 11 sites in the United States and Canada. Using a modified Breakthrough Series model [28], PR-COIN teams meet monthly via webinar and semi-annually for face-to-face workshops to review data, share best practices, and participate in improvement science training [27]. Teams at each site test innovative ideas and processes to improve care and outcomes for children with JIA. Data collected during clinical care are submitted to a shared registry and analyzed to produce aggregate and site-specific reports to identify patterns of care associated with better outcomes.

### Project improvement team

The improvement team was based at Cincinnati Children’s Hospital Medical Center and consisted of a quality improvement consultant and clinician researchers with expertise in pediatric rheumatology, SDM and the development of decision aids, family/self-management of chronic disease, and quality improvement methods.

### Development of the decision aid

To develop JIA medication choice cards [12], the team partnered with a graphic design graduate student from the University of Cincinnati and patients, parents, and clinicians from PR-COIN. Development steps included review of existing decision aids [10, 12, 29], qualitative interviews with patients [30], parents [3], and clinicians [31] and direct observation of clinical encounters, especially encounters with conversations regarding treatment options, benefits and risks/uncertainties, mode of administration, and need for monitoring. A stakeholder panel of patients, parents, physicians, advanced practice nurses, registered nurses, educational specialists, and project staff discussed and prioritized educational needs from their perspective and contributed suggestions to address these needs via face-to-face meetings, conference calls, and internet communications. This group prioritized a focus on long-term medications that control disease activity (e.g., disease modifying anti-rheumatic drugs and biologics) over medications that are intended for short-term use, or to control pain (e.g., non-steroidal anti-inflammatory medication, joint injections, steroids, etc.) because this conversation is more challenging. Prototype issue cards were critiqued by panel members and tested using plan-do-study-act cycles [25] during outpatient clinical encounters observed by improvement team members. Feedback to guide card revisions was elicited via weekly electronic surveys of teams, discussions during webinars, and following direct observation of encounters using the cards. After 18 revisions, PR-COIN stakeholders found the medication choice cards to be acceptable for use. The improvement team also developed a one-page instruction sheet with pictures depicting the intended use of the cards, a short training video, and a pamphlet containing the content of the cards for families to take home. The cards were also adapted to an interactive electronic portable document format (PDF) to facilitate implementation at PR-COIN sites that employed electronic media for patient education in the clinic setting.

### Implementation of the decision aid

The goals of this phase of the project were to (1) identify patients with JIA facing a decision to start or switch medicine, (2) provide SDM support during visits by using the medication choice cards, and (3) measure outcomes.

**Fig. 1:**
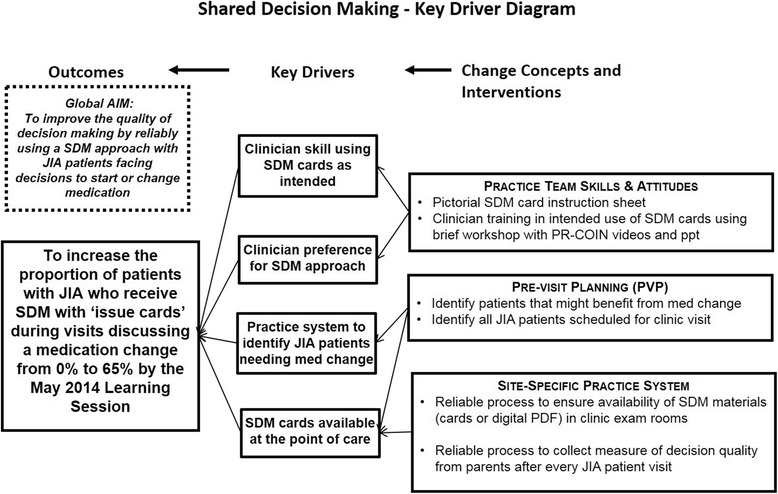
Key driver diagram

Four PR-COIN sites volunteered to use quality improvement methods to implement the SDM materials, provide feedback, administer parent surveys, and contribute data to assess SDM outcomes. Participants set a specific, measurable, achievable, realistic and timely aim [32] of increasing the proportion of patients with JIA who received SDM with JIA medication issue cards during visits discussing a medication change from 0 to 65% within three months (March to May 2014). Due to institutional review board delays at some sites, the project period was extended to early August 2014. A key driver diagram (Fig. 1) was developed to describe the relationship between the outcomes of interest, the desired characteristics of the system (the so-called “drivers”), and the structures, processes and interventions theorized to lead, via the drivers, to the desired outcomes.

A ‘train the trainer’ workshop was held at a face-to-face PR-COIN Learning Session [28] in November 2013 to enable clinician (i.e., physician and advanced practice nurses) champions from each site to train other clinicians on the intended use of the cards. Clinicians were trained to present the medication choice cards to a patient and/or parent(s) during a clinic visit and ask which of the cards they would like to discuss first. It was explained that, by picking a card, the patient or parent shows the clinician what is most important to her/him and sets the agenda for discussion. For example, parents concerned about the side effects of medications may choose to discuss that first. The cards are meant to be a flexible tool to augment the conversation that clinicians have with families. Clinicians can highlight the options most relevant for the patient given the specifics of their situation. Moreover, the clinician can share their experience and preferences while eliciting what matters most to the family about the relevant options. After reviewing and discussing the cards that the family and clinician choose to discuss, they should be able to decide together which medication best matches the family’s circumstances and preferences. A supporting video demonstrating ideal use of the cards was developed and shared with sites.

Process maps produced by each site demonstrated how SDM and the collection of outcome measures could be implemented into clinic flow processes using existing staff. The PR-COIN teams conducted iterative plan-do-study-act cycles to (1) identify all JIA patients scheduled for a clinic visit and those that might benefit from a medication change, (2) establish reliable processes to ensure the availability of SDM materials in exam rooms, (3) collect SDM outcome measures, and (4) increase clinician skill in using medication choice cards as intended.

### SDM outcome measures

Outcome measures included the three-item CollaboRATE scale, the 4-item SURE measure (Sure of myself, Understand information, Risk-benefit ratio, Encouragement), and three items developed by the improvement team to assess eligibility for use of the SDM issue cards and, if eligible, fidelity of use. The CollaboRATE scale (0–9 scale on each item, with higher scores indicating more clinician effort to engage and involve the parent) is a validated measure of SDM that is easily understood and accepted by respondents [33] and has good discriminant and concurrent validity, intra-rater reliability, and sensitivity to change [34]. The SURE measure (response options of “yes” and “no”) is a validated patient/parent report screening version of the Decisional Conflict Scale [35, 36]. A response of “yes” to all 4 items indicates no uncertainty.

### Data collection procedures

Data collection was embedded in the delivery of routine clinical care. A nurse or medical assistant responsible for discharging the patient from the clinic distributed a voluntary, anonymous, paper survey to parents after every encounter with a patient with JIA. The first question on the survey asked parents if starting or switching medication was discussed at the visit. For parents who answered “no” to this question, the survey ended. Parents who answered “yes” to this question were further asked if their child’s clinician had shown them the issues cards (pictured below the question) during the visit. If so, the final question assessed fidelity of issue card use by asking the parent if their child’s clinician asked her/him to pick the first card to discuss [8]. Each parent who received a survey was asked to place their completed survey in a collection container near the clinic exit. One PR-COIN site also tested electronic distribution of the survey where an administrative assistant for the practice sent a hyperlink for the electronic version of the survey to each parent by email or text message. To enable assessment of the completion rates achieved by the various survey collection methods, two of the four sites that collected data intermittently reviewed the clinic schedule and each patient’s medical record to determine a count of JIA patients seen on days surveys were distributed.

Clinician impressions regarding the benefits and barriers to use of the issue cards were documented during the final debriefing call with the lead clinician from each participating site.

### Data analysis

Completion rates were calculated by dividing the number of parent-completed surveys by the total number of JIA parents seen that day. The proportion of eligible visits with use of issue cards was calculated by dividing the number of parents who reported “yes” to having a been shown the issue cards by their child’s clinician by the total number of parents who reported “yes” to having a discussion about starting or switching medication. Fidelity to intended use of the cards was calculated by dividing the number of parents who reported “yes” to having been asked to pick the first card to discuss by the total number of parents who were shown the cards by their child’s doctor.

For the experience of SDM, we calculated the proportion of parents with a top score (9 s on all 3 items) on the CollaboRATE scale. For the experience of uncertainty, we calculated the proportion experiencing no uncertainty (“yes” on all 4 items) on the SURE scale. We used chi-square tests to compare eligible visits with and without use of the cards on the experience of SDM and uncertainty.

### Ethics, consent and permissions

We conducted this project in compliance with the research ethics standards of the United States of America. The teams at Cincinnati Children’s Hospital Medical Center and the four PR-COIN sites that collected data each received authorization from their local institutional review boards to conduct the study. At each location, the boards determined that the project did not involve the use and/or disclosure of protected health information and waived the requirement to obtain documentation of informed consent.

## Results

Six medication choice cards were created to enable clinicians and patients/parents to efficiently discuss medications that are reasonable options in a given clinical context (available at https://www.cincinnatichildrens.org/-/media/cincinnati%20childrens/home/service/j/anderson-center/evidence-based-care/decision-aids/arthritis%20medication%20choice%20cards.pdf?la=en and through the PR-COIN website https://pr-coin.org). Using plain language and pictorial representations, each card covered a key issue on which potential medications differ. The topics addressed by the cards were how often each potential medication must be taken, how soon the medications take effect, side effects, cost, how long each medication must be taken, and other things to consider before starting or while taking each medication.

Following implementation of the medication choice cards by the four PR-COIN sites, we collected 223 parent surveys over 18 weeks of data collection. We assessed completion rates during 7 of the 15 weeks that paper surveys were collected. During these weeks, we collected 103 surveys after 226 visits with patients with JIA, for a completion rate of 45.6%. During the 2 weeks that one site tested electronic administration of surveys by sending a link to the parent’s email account, we collected 9 surveys after 64 visits (14% completion rate). During the one week that one site tested electronic administration of surveys by sending a link to the parent by text message, we collected 2 surveys following 29 visits (7% completion rate).

During the study period, the decision aid cards were used in a median of 35% of visits where starting or switching medication was discussed. The cards were used as intended (parent was asked to pick the first card to discuss) in a median of 68% of visits where cards were used.

There were a total of 171 surveys from parents who reported that the visit involved a discussion about starting or switching medicine. The vast majority of these parents reported high levels of SDM on the CollaboRATE scale (i.e., 9 s on all 3 items) following visits with (64/76 = 84%) and without (80/95 = 84%) use of the issue cards (*p* = 1). Similarly, the vast majority of parents reported no uncertainty on the SURE scale (i.e., “yes” on all 4 items) following visits with (74/76 = 97%) and without (91/95 = 96%) use of the issue cards (*p* = 0.58).

Comments from clinicians documented during the final debriefing call with the participating PR-COIN sites were generally positive (Table 1). Reflection on facilitators of successful use of issue cards was buy-in from clinicians on the value of SDM, training and familiarity in use of issue cards, and reminders and prompts for card use and ready access at time of discussion of medication choice. Pre-clinic huddles contributed to higher rates of survey distribution and collection.

**Table 1:** Comments from clinicians

If the goal was to get the patients more engaged and involved then it definitely did that. One family actually said to me, “wow you are actually showing me choices”
We did get a lot of really good feedback. We used the ones on the computer. Because it is a shared clinic space. The families did really like them.
The cards take a little longer as you discuss medications that you are not going to use at that visit. But the thing is that parents want to know, and they ought to know for it to be part of their decision.
This may be a culture change, opening up medication choices for discussion and being less directive.

## Discussion

We sought to develop and reliably implement a decision aid to facilitate SDM between clinicians, patients with JIA, and their families concerning medication choices for arthritis. We co-produced a decision aid that met the needs of stakeholders and is publicly available for use [37]. However, in surveyed situations where use of the decision aid might have been appropriate, use occurred only one-third of the time. Efforts to embed decision aid use as part of medication discussions between patients and clinicians in routine clinical care fell short of our a priori goal (65%).

Reliable implementation of decision aids remains elusive. Our rate of 35% is similar to that seen by Group Health in Seattle over the first year during their recent decision aid implementation project for knee and hip surgery among adults [17]. In that study, however, decision aids were mailed to the home, rather than being the subject of an interactive exchange at the point of care. Our rate was less than half that reported by Canadian Cystic Fibrosis Centers that implemented a decision aid for lung transplantation [38]. The differences in the levels of reliability achieved in these implementation efforts likely reflect differences in clinical contexts, clinician training, capacity for quality improvement, resource allocation, and methods of outcome assessment. For example, this initiative was one of the first attempted interventions in the PR-COIN cohort of centers, which were gaining new experience with quality improvement methods. Therefore, the low rate of implementation may reflect insufficient quality improvement capacity at the time of the study rather than an intrinsic problem with the SDM cards. In regard to methods of outcome assessment, Arterburn et al. [17] relied on documentation that the clinician ordered the decision aid for their patient and Stacey et al. [38] relied on retrospective self-report of clinicians about their own use of the decision aid with patients. Certainly, the latter method has been shown to overestimate performance markedly across a number of settings [39]. It is possible that our measurement, based on patient/parent report, was an underestimate since the result was based on the screening item “did you discuss starting or switching medicine to treat your child’s arthritis” and didn’t specify the type of medicine. This distinction is important because the issue cards focus on discussion of disease modifying anti-rheumatic drugs and biologics, and don’t include other classes of medications, such as NSAIDs, joint injections, or steroids etc., which may have been the focus of their discussion about starting or switching medicine and for which the issue cards would not have been applicable.

Clinicians who chose to use the cards largely used them as intended. Two-thirds of parents reported that their clinician allowed them to pick the first card to discuss. One-third did not. Given the limitations to our measurement strategy, we are unable to comment on how the cards were used during these visits. It is possible that our ‘train the trainer’ approach was inadequate. We did not track the number of clinicians at each site that were exposed to the training. In hindsight, more rigorous training with knowledge assessment completed by all site clinicians, including fellows in training, may have helped uptake. Alternatively, some clinicians may have been trained but felt uncomfortable relinquishing control over the discussion agenda. Regardless of the reasons that underlie this phenomenon, past studies that video-recorded encounters show that clinician decision aid usage often lacks fidelity to intended use [40].

Embedding outcome assessment in routine clinical care also proved challenging. Completion rates were low by all methods we attempted. Distribution of a paper survey after each clinic visit performed best but yielded a completion rate of only 45%. This approach faced multiple challenges. We do not know if the surveys were distributed as intended; it was an added task competing for staff attention in a busy clinical setting. Even if distributed by staff, patient families may have been eager to leave clinic at the end of their encounter and chose not to complete the survey. Parents who didn’t face medication decisions may have been less likely to complete and return the survey. Certainly, surveying only those parents who experience the decision of interest could be more efficient, but this approach creates new challenges related to accurate and reliable case ascertainment.

Our parsimonious approach to measuring the outcomes of SDM and uncertainty failed to detect a difference between visits with and without use of the decision aid. Even though it is possible at participating sites that high levels of SDM and low levels of patient uncertainty happen without decision aids, it would be counter to observational research that has shown limited SDM in JIA visits [41]. Furthermore, systematic reviews have demonstrated low levels of SDM across a range of settings during ‘usual care’ visits that do not involve a decision aid [42] and efficacy of decision aids to improve outcomes [14]. While ceiling effects may have occurred with the measures, this was not experienced in adult healthcare settings where the measures were validated. Perhaps parents did not feel comfortable providing less than the highest score. We attempted to guard against social desirability response bias and/or concerns about loss of confidentiality by making the survey anonymous, but collecting surveys in the clinic setting may increase this threat. When we attempted to mitigate this possibility by distributing surveys electronically, our completion rate plummeted. Testing additional approaches to assess SDM outcomes is warranted.

Our project was limited by the small number of participating sites, the short 18-week data collection period, the absence of patient characteristics collected on our anonymous survey, and the observational design. Future studies could address these limitations by expanding the number of sites, extending the length of data collection, collecting more information to characterize the sample, and utilizing a randomized controlled design. Future mixed methods studies might lead to a better understanding of the barriers to implementation experienced at each site. Nonetheless, our findings make a meaningful contribution given the paucity of research on implementing decision aids and assessing outcomes related to SDM as part of routine clinical care [16].

## Conclusions

Although user acceptability of the decision aid was high, reliable implementation in routine clinical care proved challenging. Clinician leaders at each site expressed enthusiasm for using the decision aid, but it is unclear how broadly this enthusiasm was shared by their colleagues. We further reason that what appears to be a shortcoming of implementation may not reflect an intrinsic problem with the SDM cards, but rather it may be attributed to the quality improvement inexperience of the network at time of the study. Our parsimonious approach to outcome assessment failed to detect a difference between visits with and without use of our decision aid.

Our next steps include revising the JIA medication choice issue cards based on ongoing stakeholder feedback and to reflect medication changes (e.g., new modes of administration, new products). Periodic training of new clinicians and centers entering the network and sharing of best practices on implementation will continue in the context of PR-COIN to promote spread of SDM. Our findings raise interesting questions for future research. Might an electronic format that is enabled by decision support from the electronic health record engender more reliable use? If SDM represents a culture change in the practice of rheumatology, are additional tools needed to facilitate this process with a broader range of decisions faced by children with JIA (e.g., pain relief, etc.)? Could the burden of outcome measurement be lessened by leveraging the infrastructure that is used to collect patient reported outcomes and satisfaction? Indeed, it appears there are miles to go before reliable implementation of decision aids becomes a reality in routine clinical practice [16, 33]. Innovative approaches are needed to facilitate use of decision aids and assessment of outcomes.

## Abbreviations

JIA: Juvenile idiopathic arthritis
PDF: Portable document format
PR-COIN: Pediatric rheumatology care and outcomes improvement network
SDM: Shared decision making
SURE: Sure of myself, understand information, risk-benefit ratio, encouragement
